# Rxnat: An Open-Source R Package for XNAT-Based Repositories

**DOI:** 10.3389/fninf.2020.572068

**Published:** 2020-11-09

**Authors:** Adrian Gherman, John Muschelli, Brian Caffo, Ciprian Crainiceanu

**Affiliations:** Department of Biostatistics, Johns Hopkins Bloomberg School of Public Health, Baltimore, MD, United States

**Keywords:** XNAT, R, neuroimaging, nitrc, connectome, MRI, normalization, neuroconductor

## Abstract

The extensible neuroimaging archive toolkit (XNAT) is a common platform for storing and distributing neuroimaging data and is used by many key repositories of public neuroimaging data. Some examples include the Neuroimaging Informatics Tools and Resources Clearinghouse (NITRC, https://nitrc.org/), the ConnectomeDB for the Human Connectome Project (https://db.humanconnectome.org/), and XNAT Central (https://central.xnat.org/). We introduce Rxnat (https://github.com/adigherman/Rxnat), an open-source R package designed to interact with any XNAT-based repository. The program has similar capabilities with PyXNAT and XNATpy, which were developed for Python users. Rxnat was developed to address the increased popularity of R among neuroimaging researchers. The Rxnat package can query multiple XNAT repositories and download all or a specific subset of images for further processing. This provides a lingua franca for the large community of R analysts to interface with multiple XNAT-based publicly available neuroimaging repositories. The potential of Rxnat is illustrated using an example of neuroimaging data normalization from two neuroimaging repositories, NITRC and HCP.

## 1. Introduction

Medical imaging research is constantly evolving, which led to a dramatic increase in the availability, scale, and number of publicly-available image datasets. These datasets are heterogeneous, which raises substantial challenges for central organization and harmonization. Indeed, even downloading of relevant data from a number of different repositories is challenging because it requires multiple manual steps, which render the process essentially irreproducible. Moreover, repetitive downloading of datasets with different characteristics substantially increases the effort of the potential users.

Various imaging data hosting platforms have recently emerged, with the most prominent being the eXtensible Neuroimaging Archive Toolkit, XNAT (https://www.xnat.org) (Marcus et al., 2007). XNAT is an open source imaging framework for neuroimaging informatics, which is structured as a database and contains procedures for storing, downloading, and querying the imaging data, as well as managing user level access permissions. Notably, XNAT has become a standard for the database backbone for dissemination of large public imaging repositories. Some examples of large image repositories that rely XNAT framework are (for more examples see https://www.xnat.org/about/xnat-implementations.php):

- **NITRC** (https://nitrc.org/). Neuroimaging Informatics Tools and Resources Clearinghouse is currently a free one-stop-shop collaborative resource for researchers who need neuroimaging analysis software, publicly available data sets, or computing power (Kennedy et al., 2016).
- **ConnectomeDB** (https://db.humanconnectome.org/). The Human Connectome Project (HCP) is designed to construct a complete map of the structural and functional neural connections *in vivo* within and across individuals (Van Essen et al., 2013).
- **XNAT Central** (https://central.xnat.org/). XNAT Central is a database for sharing neuroimaging and related data with select collaborators or the general community (Herrick et al., 2016).

Most end users interact with XNAT via a graphical user interface (GUI), which enables researchers to download images. This interaction is manual and requires multiple point and click actions that substantially slow down image processing pipelines. Using a GUI can be difficult and time intensive, especially if a large number of images is required or multiple XNAT servers need to be queried. Thus, a software-based approach is necessary to eliminate the friction induced by the manual interaction with XNAT. Given the increased complexity, size, and number of projects that use XNAT, using software to interact with XNAT-based repositories can save time, improve workflow efficiency, and increase analytic reproducibility. Indeed, having public software that describes the data downloading process is more transparent and less prone to errors than describing the inclusion/exclusion criteria. Moreover, software can easily be adapted to extract different datasets, which substantially increases analytic efficiency.

We developed the package Rxnat (https://github.com/adigherman/Rxnat) to interface with XNAT databases in the R programming language (R Core Team, 2017). The package (1) authenticates the user, (2) queries and extracts information, and (3) downloads images and other data from these databases. The package enables users to navigate heterogeneous neuroimaging datasets using a unified syntax based on standard R data structures. We demonstrate the utility of Rxnat by describing the associated code and including an example of combining images from multiple sources in an analysis. The program has similar capabilities with PyXNAT (Schwartz et al., 2012) and XNATpy (Achterberg, 2015), which were developed for Python users. Rxnat is thus designed for a different analytic community than PyXNAT/XNATpy. It also interacts with the various analytic R packages in Neuroconductor (https://neuroconductor.org/) (Muschelli et al., 2018) and popular data manipulation packages such as **dplyr** (Wickham et al., 2019). Using Rxnat allows to query specific subgroups of data from multiple XNAT-based image repositories, download results data in a standard format, and perform joint analyses. Thus, Rxnat provides the infrastructure to conduct complete analyses on one platform, R.

## 2. Materials and Methods

Setting up an XNAT instance requires a server and specialized database expertise. We will not cover setting up XNAT servers here; instead, we will focus on how to interact with such servers. For more information of setting up an XNAT server, see https://www.xnat.org/.

### 2.1. R as a Complete Analytic Platform

Rxnat is an essential component of an analytic platform that starts with downloading public image data, pre-processing and analyzing it on one open source platform: R (R Core Team, 2017). An advantage of R is that it is designed specifically for data analysis and has benefited from a large community development effort. While R is not the main programming language for image analysis, its community and ecosystem are developing rapidly (Tabelow and Whitcher, 2011).

Some of the advantages of having an integrated R platform are that: (1) it offers all of the primary components of modern interpreters and is at the forefront of data science software development; and (2) R provides support for object-oriented and functional programming.

The computational time associated with neuroimaging software is of primary concern due to the large size of the imaging files and databases. Many imaging software tools are based on C++, which makes them fast and efficient. In contrast, R is a high level interpreted language that historically has been viewed as a slower alternative. However, R offers several avenues for utilizing compiled code. Most notably, the **Rcpp** package facilitates wrapping C++ code and libraries, which can create or adapt powerful and fast imaging operations. Also, R can be used as an interface and pipelining language, calling installed imaging packages from the command line, such as the **fslr** (Muschelli et al., 2015) package that calls the FMRIB Software Library (FSL) (Jenkinson et al., 2012). This interface of imaging packages is similar to the **Nipype** module in Python (Gorgolewski et al., 2011). Both R and Python pipelining are extremely useful to combine traditional pre- and post-processing analytic steps into unified analytic pipelines.

Recent efforts in data science have pushed R into the vanguard of conceptual thinking and implementation in this area. These efforts includes plotting, interactive graphics, reproducible research, data management and manipulation, dissemination and app development (Xie, 2014; Tustison et al., 2015; Wickham, 2016; Sievert, 2020). Specifically in neuroimaging data science, recent packages have increased R's capacity for static and interactive display of neuroimaging data and its analysis (Mowinckel and Vidal-Piñeiro, 2019; Fisher, 2020; Muschelli and Gherman, 2020).

R provides a powerful package management system, which allows for a series of checks to be performed to ensure operability. The **testthat**, **RUnit** and other packages provide unit testing procedures for stability (Wickham, 2011; Burger et al., 2018). The comprehensive R archive network (https://cran.r-project.org/) is primary source of general R packages. The R package management system has also allowed for the development of domain-specific package repositories that inspire collaboration and dissemination within more tightly coupled scientific domains. Perhaps the largest such example is Bioconductor, which focuses on computational biology (Gentleman et al., 2004). Another effort is Neuroconductor, which is a domain specific R repository for image analysis (Muschelli et al., 2018). Rxnat is a component package of Neuroconductor and was developed as a core utility by the Neuroconductor developers.

For interaction with XNAT servers, R provides a number of options. The **httr** package provides an interface in R using the standard HTTP methods and verbs for interacting with RESTful APIs, similar to the **requests** module in Python (Wickham, 2019). The **RCurl** and **crul** packages provide additional functionality, with the added benefit of using the popular curl-specific syntax (Temple Lang, 2020). Thus, the packages **httr**, **RCurl**, and **crul** allow powerful queries and calls to XNAT servers.

The introduction of the **dplyr** (Wickham et al., 2019) R package allowed for more intuitive data manipulations steps within R. These steps include subsetting data on rows (filter) or columns (select), summarizing data (summarize), creating frequency tables (count), among others, all in a unified framework and data type (a data.frame). **Rxnat** allows users to query databases and return data.frames. Thus, users who are familiar with **dplyr** commands can use Rxnat directly, without the need to learn the XNAT-specific querying syntax.

### 2.2. XNAT

XNAT is an open-source imaging informatics software platform dedicated to managing and distributing neuroimaging data. The under-laying framework uses XML for both database back-end operation as well as the front-end web interface. The primary set of XNAT features handle several core tasks:

- **Organize and Share Data** - Data stored in XNAT is associated with user defined projects and allows giving access to users on a project-by-project basis.
- **View and Download Data** - XNAT includes an online image viewer that supports a number of common neuroimaging formats, including DICOM and Analyze.
- **Upload Data** - XNAT offers a variety of methods to upload data including image and metadata importing directly from scanners, customized upload forms, and ZIP enabled uploaders.
- **Securing Access to Data** - Quality control procedures provide secure ways to access the data as well as control its accessibility by fellow researchers and by the general public.
- **Search and Explore Data Sets** - XNAT provides a web interface that allows users to store, retrieve, navigate, and query the imaging data.
- **Process Data** - XNAT includes a powerful pipeline engine that allows the programming of complex workflows with multiple levels of automation.

The Rxnat package is focused on query and data download as these two operations are most commonly used by brain imaging researchers looking to access XNAT imaging data. Here we focus on the download capabilities of Rxnat, though future releases will expand its upload capabilities.

### 2.3. Integration With Neuroimaging in R

#### 2.3.1. Architecture and Design

The Rxnat package relies on the R packages **httr**, **RCurl**, and **crul** to interact with an XNAT server. The XNAT REST API structure has a collection of resources that give access to both files (e.g., brain images) and metadata. Although the resources can be accessed through the XNAT search engine individually, running complex queries on aggregated data and downloading a certain subset of images/metadata is not possible natively (Marcus et al., 2007). Using the Rxnat package functionality, a researcher will be able to combine multiple XNAT datasets, filter the aggregated data based on multiple criteria and download the results/images to be used in analysis pipelines.

## 3. Examples

### 3.1. Extracting Demographic Information From Multiple Image Repositories

We show how to use the Rxnat package to select study participants aged 26–40 from both NITRC and HCP image repositories and download their magnetic resonance imaging (MRI) scans. Images are further processed in R using inhomogeneity correction, brain extraction, and tissue-class segmentation. Results of this processing are shown for one study participant for illustration purposes. Intensity normalization is then applied to each image and intensities distributions are compared before and after intensity normalization. The complete R code for this example can be found here: https://raw.githubusercontent.com/adigherman/Rxnat/master/paper_code.R.

#### 3.1.1. Connect to NITRC and HCP

The first step is to connect to each image repository using the xnat_connect function. Authentication is done using usernames and passwords. These values can be included in the command as username and password pairs. Alternatively, specifying xnat_name indicates which environment variables to use for authentication. For example, with the nitrc object below, as xnat_name is set to “NITRC”, Rxnat will look in environment variables NITRC_RXNAT_USER and NITRC_RXNAT_PASS for authentication. This allows the code to be shared without revealing credentials and authentication.

**Figure:**
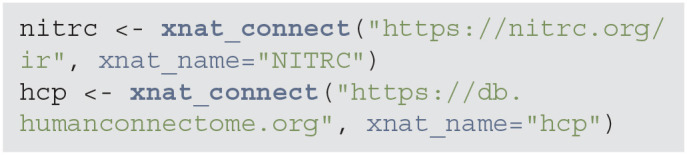


The result of the xnat_connect function is an object of class RXNATConnection. We decided to use objects to store return query data as this will substantially reduce the time for subsequent operations and would not require to re-query the XNAT server. The object will not store images or other large data, but will facilitate the creation of subsequent Rxnat calls to download the data. The methods are classified into two main categories:

1. Internal methods required for keeping track of certain connection parameters as well as being able to perform internal operations/sanity checks on an RXNATConnection object.
  - base_url - returns the connection base URL and is used to create fully fledged URLs to download one or multiple images/resources.

**Figure:**
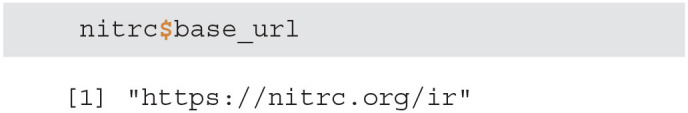
  - xnat_name - prints the name of the XNAT this objects is connected to and is used internally to create the name of the system environment variable that holds the username and password for authentication (in case the credentials are not passed as arguments when initializing an XNAT connection).

**Figure:**
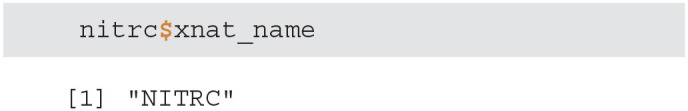
  - jsid - outputs the JSESSIONID - a session identifier for the connection. This unique temporary identifier is used as an authentication token for all calls to the XNAT server.

**Figure:**
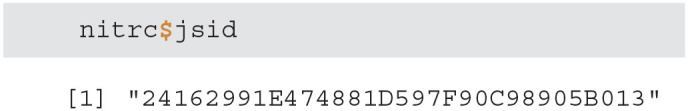
  - close - clears the JSESSIONID variable and closes the XNAT connection

**Figure:**
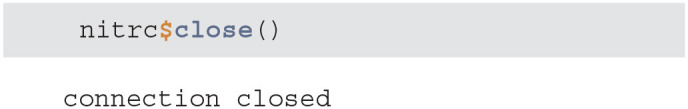
  - is.connected - checks the connection status signaling if a new connection needs to be established.

**Figure:**
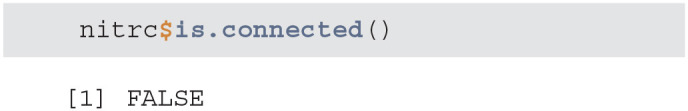
2. Usability functions are the external facing Rxnat functions that allows users to interact with an XNAT repository by querying, filtering and downloading data.
  - projects - returns a tibble listing all XNAT projects that are accessible based on the supplied user credentials. This could potentially be only a subset of all projects hosted on

**Figure:**
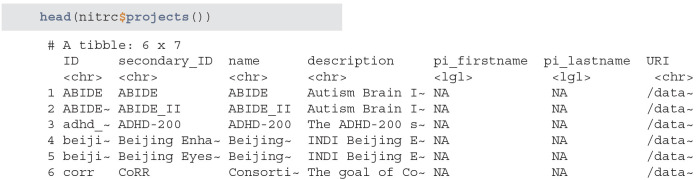
    the XNAT server as privileges sometimes are granted on a per project basis.
  - subjects - outputs a tibble listing all available subjects from all accessible projects. The listing also includes basic clinical information.

**Figure:**
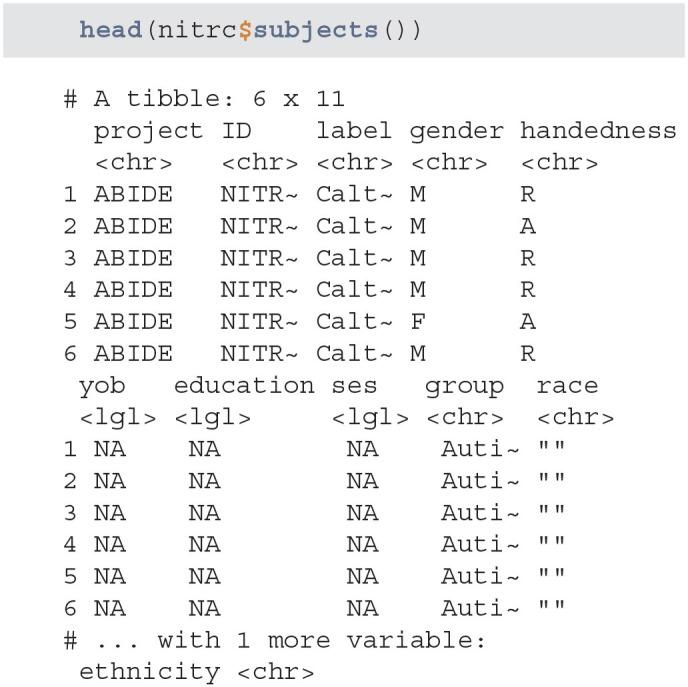
  - experiments - returns a tibble listing all of the available experiments. The list is retrieved based on the provided user credentials and only lists the experiments associated with studies that are accessible.

**Figure:**
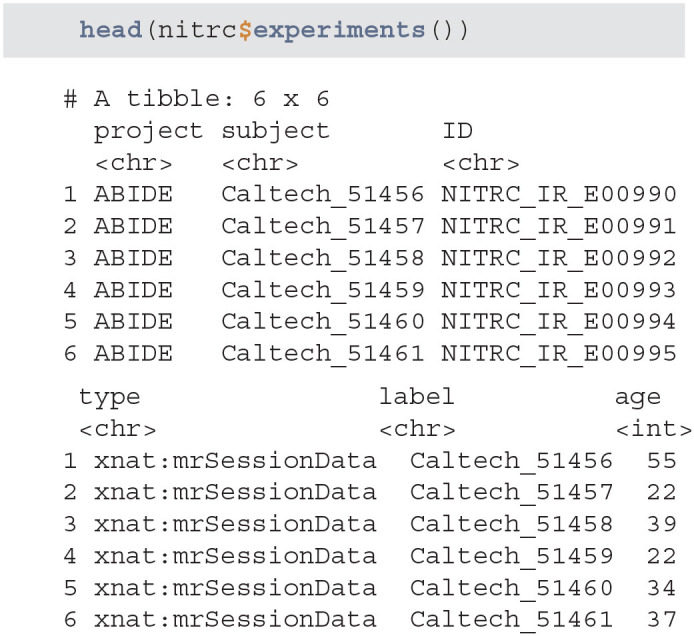
  - get_xnat_experiment_resources - returns a tibble with all the resources (files) associated with a particular experiment. The listing will provide information on the type of resource, size information as well as a URI (Uniform Resource identifier) to allow direct downloads of selected resources.

**Figure:**
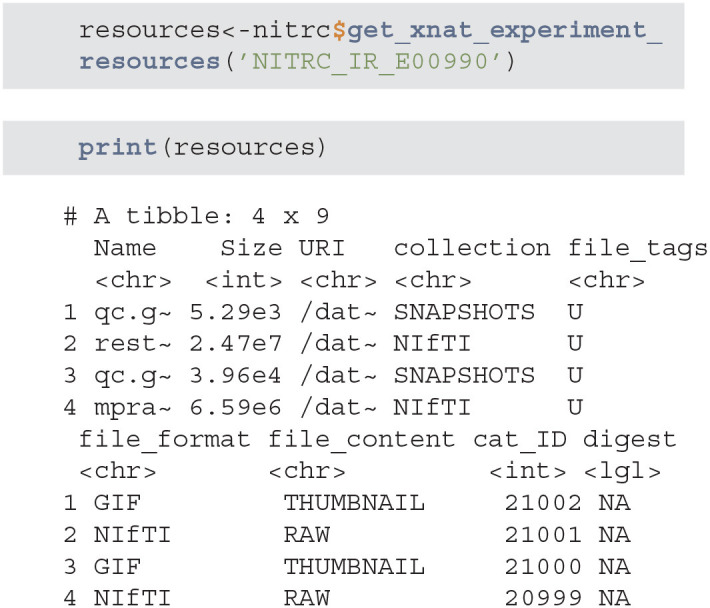
  - download_file - downloads a single resource file. As an example, we will use the resources tibble from the previous method example to download the NIfTI MP-RAGE RAW image.

**Figure:**
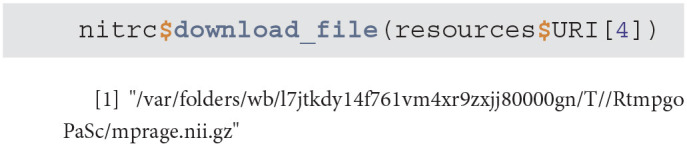
  - download_dir - downloads multiple resource files at the same time

**Figure:**
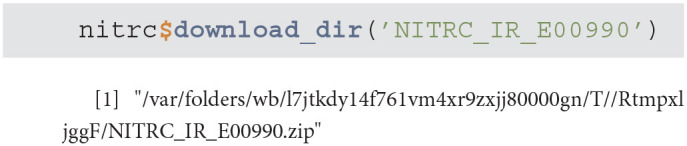
  - scans - is used to filter the XNAT repository data and return a subset of subjects that match specific criteria. As an example we will query all NITRC projects for all subjects aged 26 that have at least one T2 type image.

**Figure:**
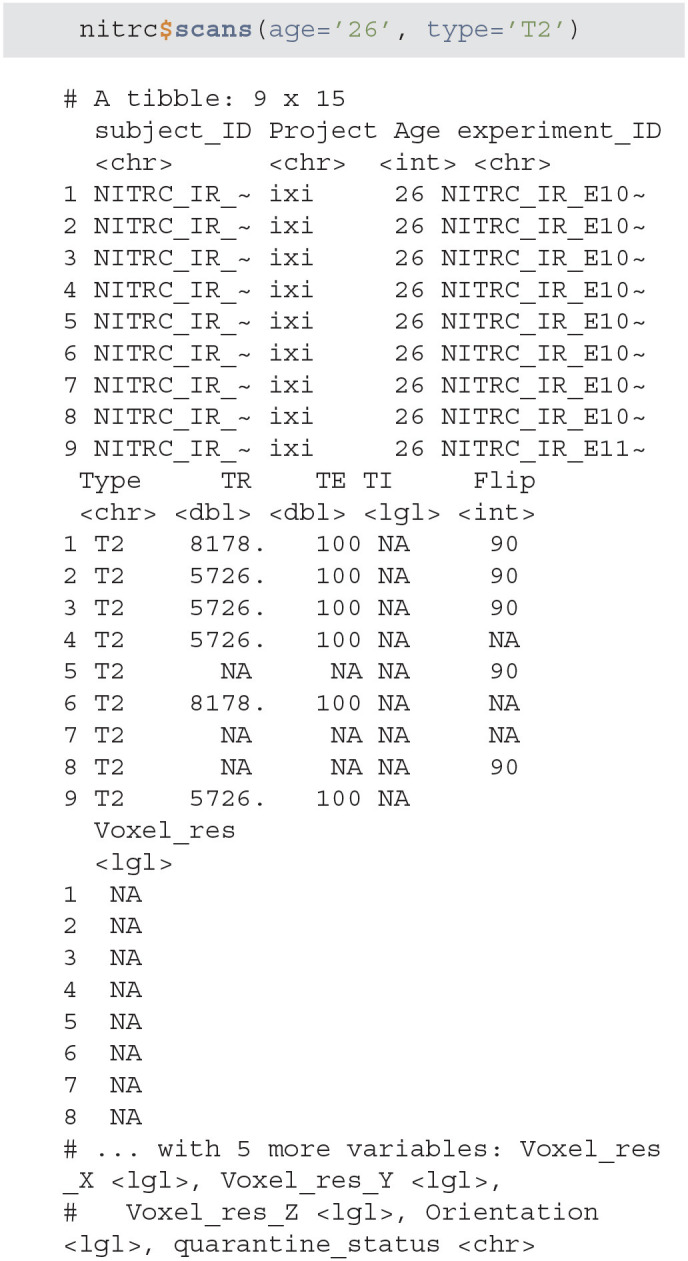

The amount of information contained in an XNAT server can be massive, so it is important to try to optimize the query/download time. An object of class RXNATConnection stores the projects, study participants, experiments, and scans query results internally, which substantially speeds up subsequent query operations. Thus, the initial connection call to an XNAT resource collects and stores some of the quickly retrievable data in the return object. This information is then used in every subsequent call without re-querying the XNAT server.

#### 3.1.2. Query and Download Subject Data

Next, we query the NITRC and HCP repositories and acquire all patient data with the subjects method. We will then aggregate the data into one data.frame by appending the data by row:

**Figure:**
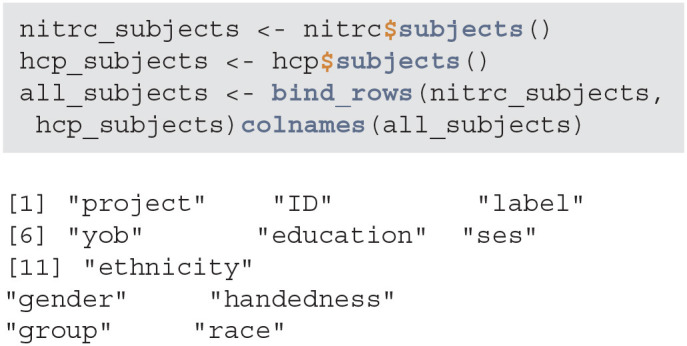


Data seems to have information on certain demographic variables. However, a number of these variables are missing or may not be captured for this project:

**Figure:**
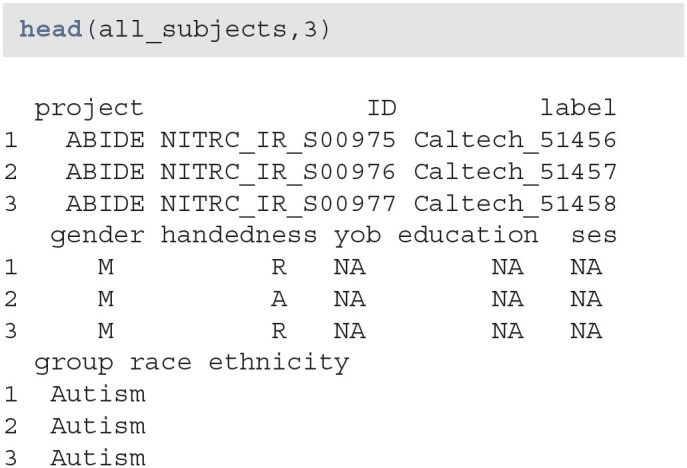


When creating subsets of data based on querying individual variables different rules may be necessary, especially when aggregating multiple studies or projects. Also, some demographic variables are returned from XNAT using that query (such as age), that are not encoded in the subject-level data. Thus, using a combination of both queries and aggregate data may be necessary. This may be surprising, but in longitudinal studies some variables change over time (e.g., age, BMI, smoking status) whereas others (e.g., handedness) do not. Thus, the time-varying variables may be stored at the scan-level while the time-invariant variables may be stored at the subject-level.

#### 3.1.3. Extract Experiment Data

For the purpose of this example, we focus on T1-weighted images, which are widely used for brain image segmentation. To retrieve the list of all experiments that have an associated T1 image, we use the scans function. NITRC stores T1 images under the T1 label, while HCP stores them under the T1w label. The data set is augmented to include the data source and image sequence, which is especially useful when combining multi-sequence data.

**Figure:**
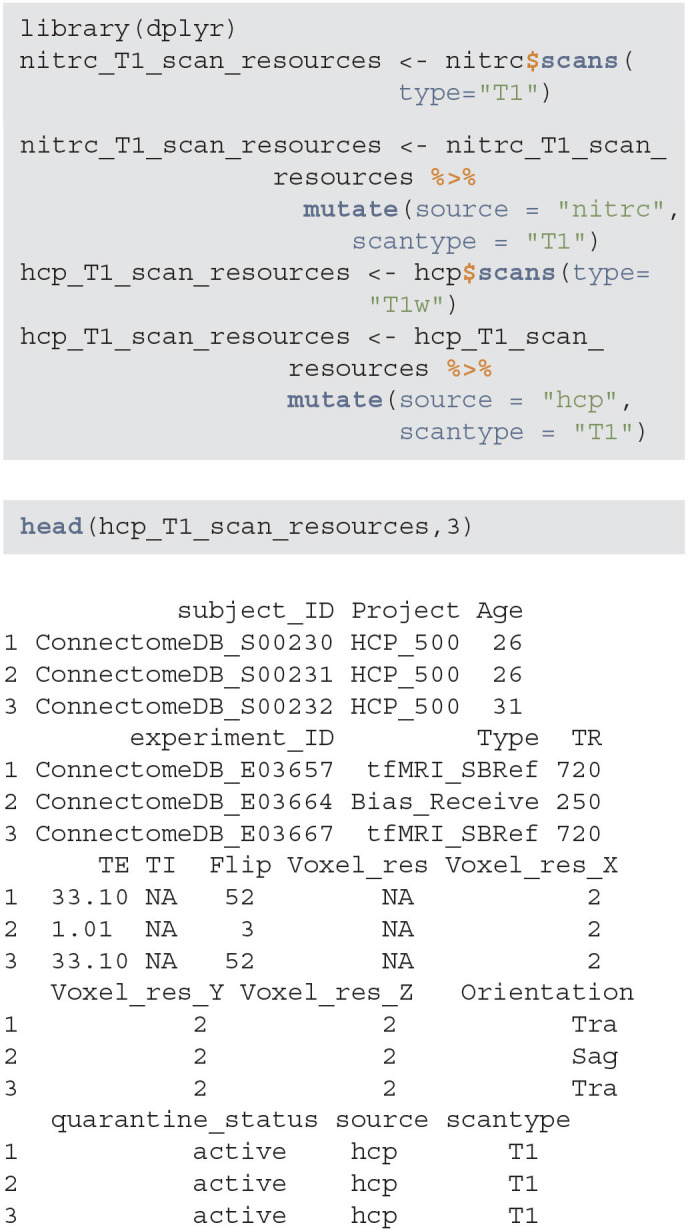


The nitrc_T1_scan_resources and hcp_T1_scan_resources data frames contain information associated with T1-weighted images available in the NITRC and HCP repositories, respectively. This information can be combined and then used to select the sample of interest. However, some studies may have different data encoding procedures. For example, some studies may collect age whereas other studies may collect the date of birth and visit date instead. Therefore, it is important to harmonize the common fields before starting the manipulation of the joined data.

#### 3.1.4. Filter Results to Select a Subgroup

The next step is to aggregate the NITRC and HCP T1 resources/image information, join them with the subjects information, and filter for study participants with age between 26 and 40. Below T1_resources data frame combines the information about T1-weighted images with the subject-level data contained in the all_subjects data frame. The data frame age_26_to_40_group now contains all the necessary information to extract the specific subsample of interest.

**Figure:**
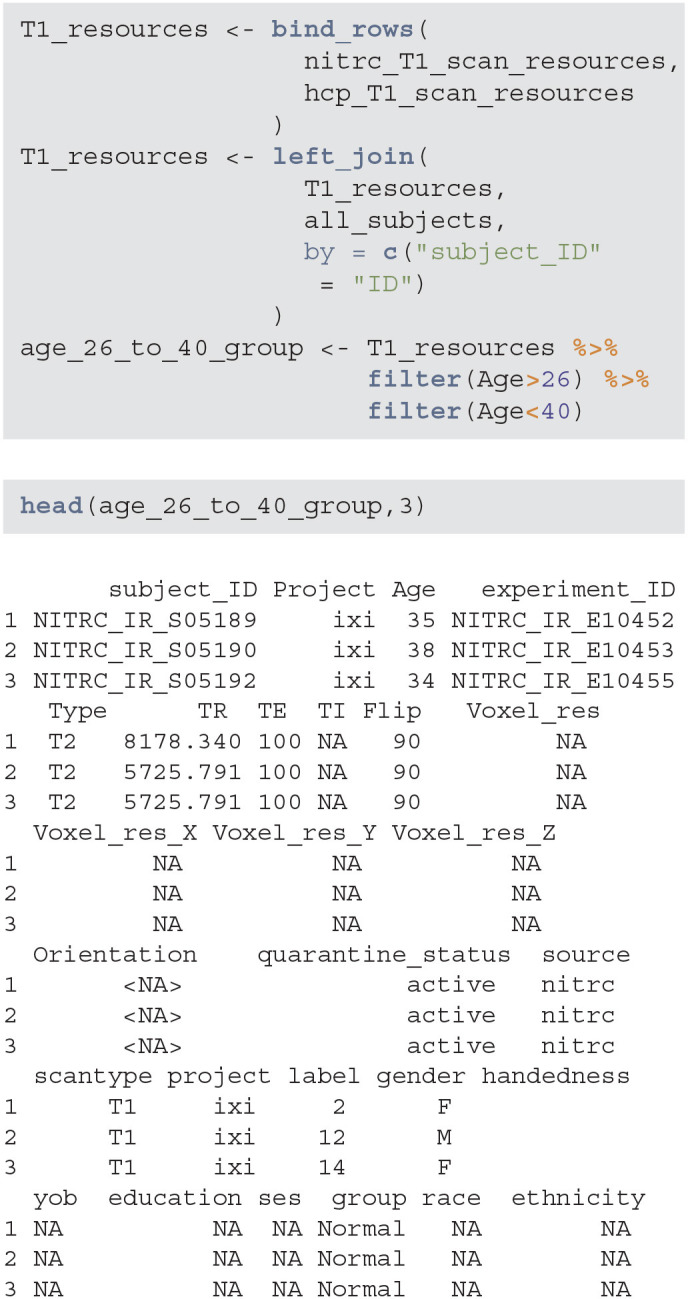


Using these data it is now easy to produce (Table 1), which provides the gender distributions for the NITRC and HCP populations of 26- to 40-year-olds. In this age group NITRC has a higher percentage of male participants (56%) compared to HCP (34%). In addition to demographic and clinical information, scanning parameters may be wildly different across studies, and correction or adjustment of these differences may be necessary for analysis.

**Table 1:** Gender distribution in the NITRC and HCP repositories for study participants between 26 and 40 years of age.

**XNAT server**	**Total subjects**	**Male**	**Female**
**Gender distribution age 26–40**			
NITRC	132	75 (56%)	60 (44 %)
HCP	410	138 (34%)	272 (66%)

#### 3.1.5. Image Processing Pipeline

Supplementary Figure 1 displays the T1-weighted image from one of the study participants. The image still contains much of the mouth, neck, and brain stem, which need to be removed before applying standard imaging processing tools, such as brain segmentation. We illustrate our processing steps on this image, but the process is applied to the entire data set.

**Figure 1:**
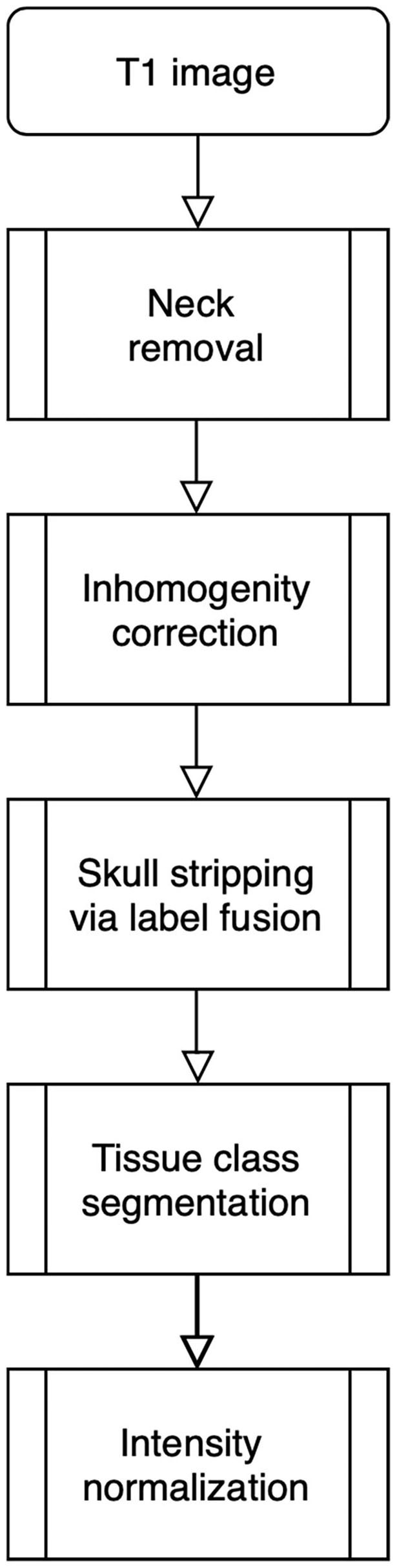
Image processing pipeline: neck removal, inhomogeneity correction, skull stripping via registration and label fusion, tissue class segmentation, and intensity normalization across studies.

The processing pipeline shown in Figure 1 will read and reorient the original T1 image, perform registration-based neck removal, brain extraction using a form of multi-atlas label fusion (MALF) (Wang et al., 2012) brain segmentation and tissue class segmentation using the FAST algorithm (Zhang et al., 2001).

We now provide the major step of the software implementation. The first step is to download a sample T1-weighted image using the download_dir function. For this example we download an image from NITRC. The experiment_ID is a NITRC experiment identifier for a study participant between 26 and 40 years of age. The result of the download is a compressed directory, which is decompressed in a temporary location to access the files.

**Figure:**
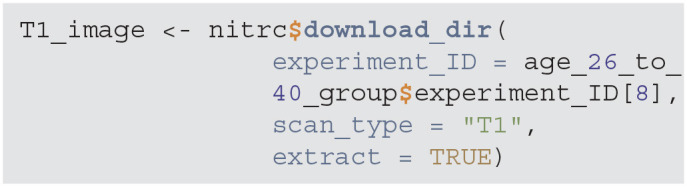


The T1_image object is a vector of files names from the temporary download folder and we will read in the 1st element which is the T1 weighted NIfTI file name.

NB: The rest of this section describes an image processing and analysis pipeline of T1 weighted structural MRI, but it is not Rxnat specific. Please feel free to skip to the section 3.1.6 if desired.

To ensure that all images have the same orientation, we use the readrpi function from the fslr package, which uses FSL to read and reorient a T1-weighted image.

**Figure:**
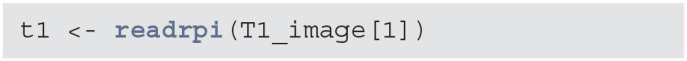


**Figure 2:**
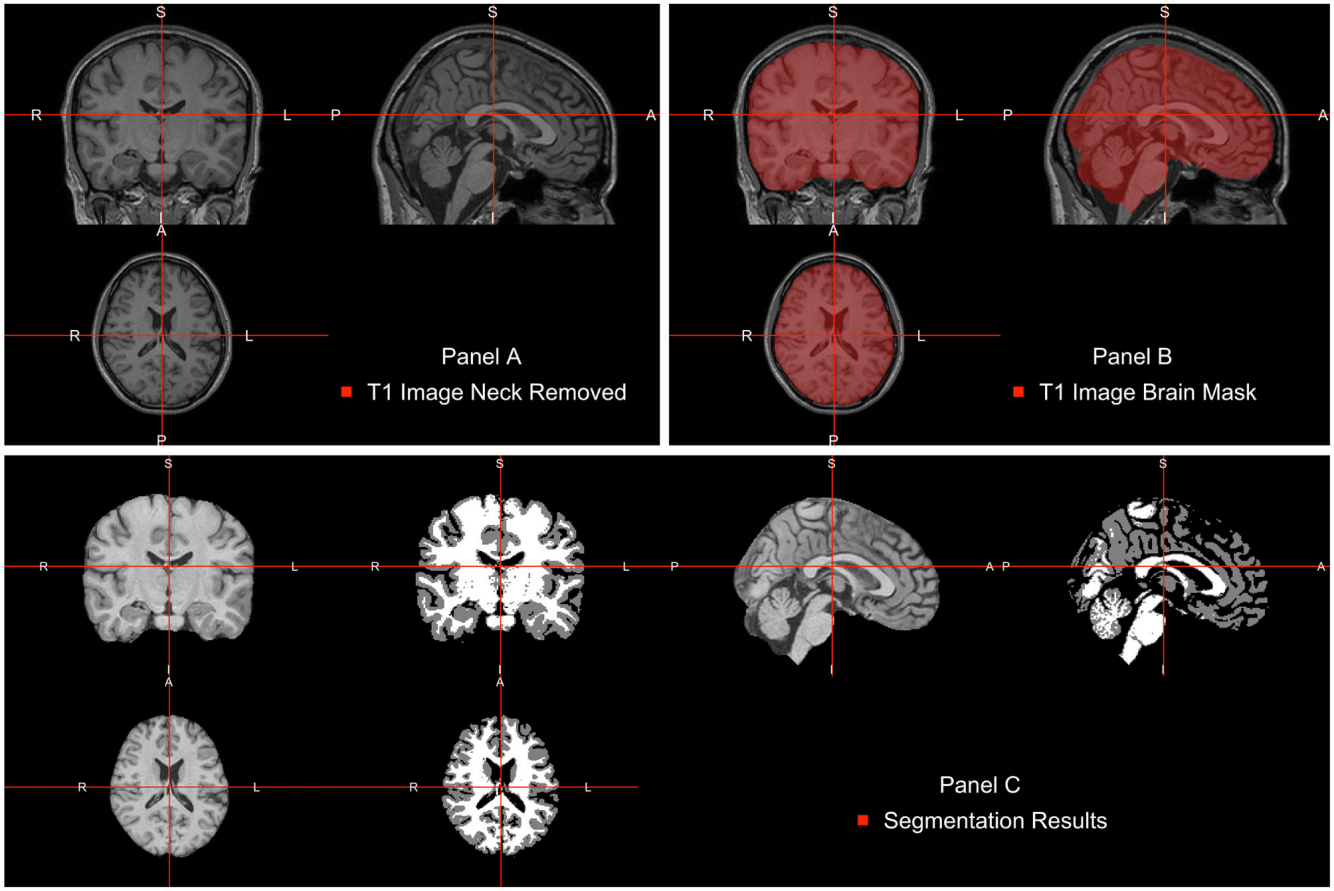
Pipeline image processing. **(A)** T1-weighted image after bias-field correction and neck removal. **(B)** Brain mask (red) estimated using multi-atlas label fusion. **(C)** Brain image plotted next to a three-class tissue segmentation in white matter (WM, color-labeled white), gray matter (GM, color labeled gray), and cerebrospinal fluid (CSF, color labeled black).

The neck removal step is implemented using the remove_neck function from the extrantsr (Muschelli, 2019) package. The empty image dimensions (including the neck slices) can be dropped by using the function dropEmptyImageDimensions from the neurobase (Muschelli, 2020) package (Figure 2A).

**Figure:**
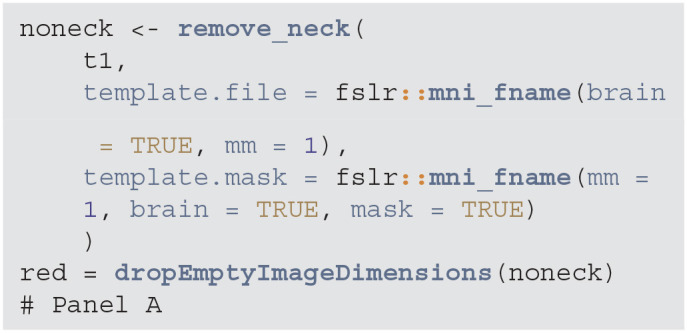


Many MRIs contain a bias field, which is a low frequency, smooth, non-biological signal introduced by magnetic inhomogeneities. To correct the bias field signal we use the bias_correct function from the extrantsr package, which uses the N4 inhomogeneity correction (Tustison et al., 2010).

**Figure:**
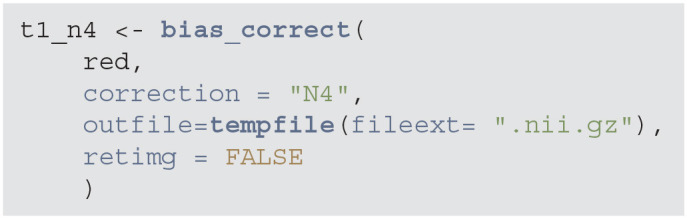


Once images are bias field corrected, we apply brain extraction using a form of multi-atlas label fusion (MALF) (Wang et al., 2012). MALF uses a collection of previously labeled brain images (atlases), aligns the T1-weighted image to each atlas, and obtains a labeled T1-weighted image for each registration. These labels are then combined, or fused, into one labeled map of the processed T1-weighted image. This approach is implemented using the malf function from the malf.templates package, which includes the templates from the 2012 MICCAI Multi-Atlas Labeling Challenge (Landman et al., 2012). Figure 2B displays the T1 image overlaid with the estimated brain mask using this approach indicating that the brain tissue is well estimated and extra-cranial areas are excluded.

**Figure:**
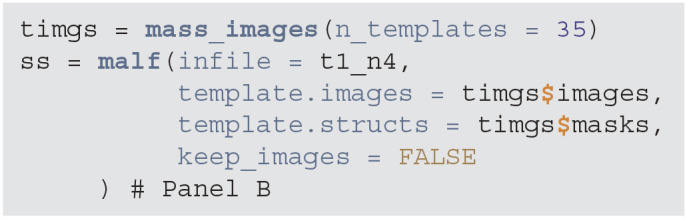


All these steps an be done with fewer lines of code using the preprocess_mri_within function from the extrantsr package. This function performs N4 bias correction, image registration (if multi-sequence data is given), skull stripping (estimating the brain mask if one is not supplied), and brain mask application to the registered images. These steps have already been applied one-by-one, save for applying the brain mask, but this wrapper is useful for doing all the steps with one function, especially in cases where you are using multi-sequence data where registration is required.

**Figure:**
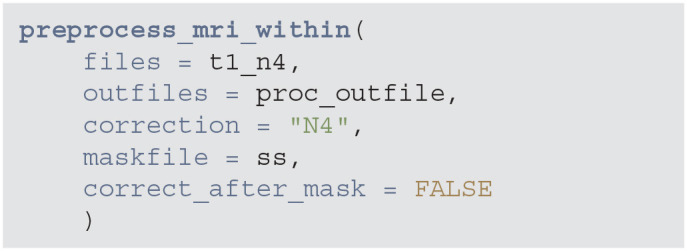


Intensity normalization is an important component of image analysis, especially when results of image processing depend on voxel intensities or when one is interested in combining intensity information across individuals. For example, when images are thresholded for low values, we may want that threshold to have the same interpretation across scans. To achieve that we need to apply one of the many methods for intensity normalization; see Reinhold et al. (2019) for a discussion.

We will use the WhiteStripe intensity normalization method introduced by Shinohara et al. (2014). This approach, estimates a small area in the tail of the T1-weighted image intensity distribution, labeled the “white stripe,” as it generally corresponds to white matter voxels. The mean and standard deviation (SD) of the voxel intensities in this area is calculated, and the image is z-scored by this mean/SD. WhiteStripe intensity normalization can be implemented using the whitestripe and whitestripe_norm functions from the WhiteStripe package.

**Figure:**
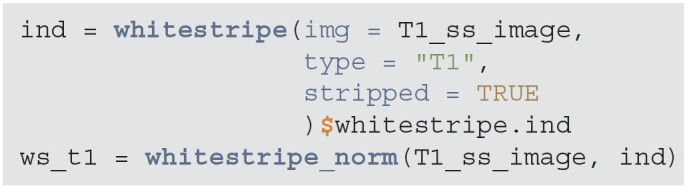


After WhiteStripe normalization, the intensities of the brain image are interpreted as standard deviations from the mean of the normal-appearing white matter (NAWM). We would like to compare the effects of intensity normalization within tissues classes. To do that, one needs to perform tissue class segmentation on each image. This is implemented here using the FAST function from FSL. FAST segments a 3D brain image into different tissue types (GM—Gray Matter, WM—White Matter, CSF—Cerebrospinal Fluid). As our images are already N4 corrected, we will use the fast_nobias from the fslr package, which assumes that the bias field was removed. Figure 2C displays the results of this segmentation.

**Figure:**
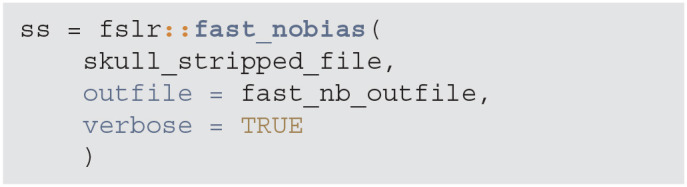


**Figure 3:**
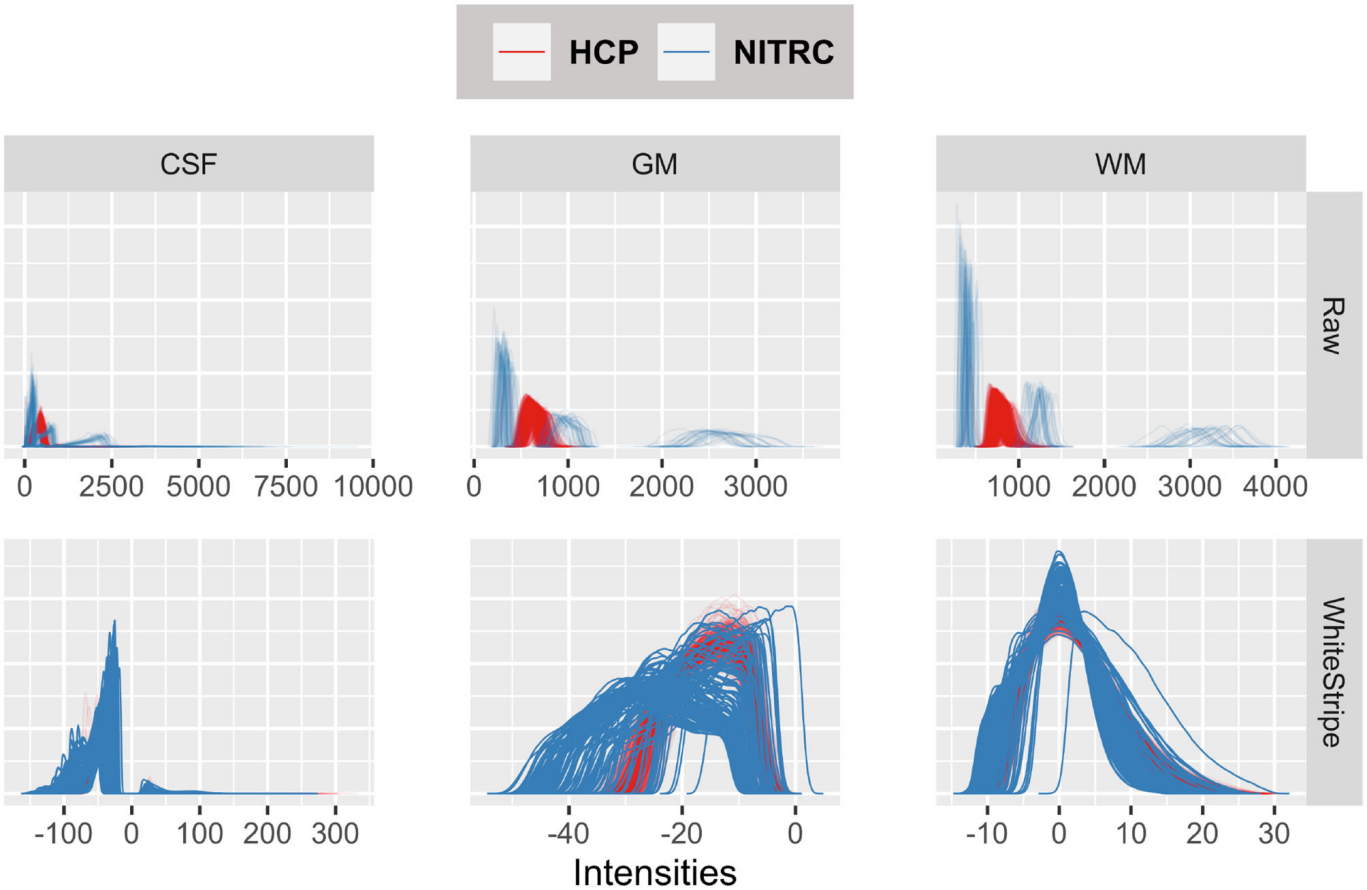
Tissue intensity densities in raw (first row) vs. WhiteStripe intensity normalized images (second row). The distribution of intensities for each study participant and tissue type is represented by one density (line) by tissue type: Cerebrospinal Fluid (CSF, **left**), Gray Matter (GM, **middle**), White Matter (WM, **right**). The density color coding corresponds to the different repositories: blue for NITRC and red for HCP.

Figure 3 displays the tissue intensity densities in raw (first row) vs. WhiteStripe intensity normalized images (second row). The distribution of intensities for each study participant and tissue type is represented by one density (line) by tissue type: Cerebrospinal Fluid (CSF, left panels), Gray Matter (GM, middle panels), White Matter (WM, right panels). The density color coding corresponds to the different repositories: blue for NITRC and red for HCP. The densities of raw intensities can be clearly separated by repository, indicating that the raw units have a fundamentally different interpretation in the two studies, even when separated by tissue classes. These substantial differences are likely due to different scanning protocols, scanner types, and scanner manufacturers. Within each study there is a higher degree of overlap of MRI voxel intensity distributions, which is likely due to the study-specific scanning protocols and machines. The WhiteStripe-normalized data (second row in Figure 3) indicates that the CSF and WM intensity distributions across all subjects and both studies are similar (see second row, right panels). For GM the overlap of distributions is much improved compared to the raw data. However, there is still separation between the studies, which may require additional normalization using, for example, RAVEL (Fortin et al., 2017).

#### 3.1.6. Easier With an Interface

The above image processing pipeline shows an analysis of data from multiple sources. Moreover, we stress that researchers should be careful with data aggregation as harmonization is almost surely required. While Rxnat was not the explicit focus, the full process, without a programmatic interface the process would be navigating and filtering based on the NITRC and HCP graphical interfaces. As the number of data sources grow, even if they are supported by XNAT, the probability of these interfaces being the same goes down drastically. Rxnat allows us to perform this data aggregation with reproducible scripts where the same interface works with multiple data sources, and demographic and clinical data can be analyzed and explored without downloading all individual data. To reiterate, the above analysis and data aggregation is possible without Rxnat, but is more difficult and not scripted.

## 4. Discussion and Conclusion

Large public data repositories have become focal points in neuroimaging research. Such resources can be used to: (1) conduct meta analyses and synthesis across studies (Yarkoni et al., 2011); and (2) provide priors for data fusion with smaller local studies. Moreover, many repositories contain information about data replicability and reproducibility (Zuo et al., 2014). Thus, they can be used to optimize processing pipelines and understand inter-subject and inter-site replicability.

Public image databases often provide direct and relevant scientific information, particularly on establishing norms. Further, public databases are core resources for methodology development in neuroimaging. With easy access to these databases, a wider variety of researchers can access, use, and test emerging methodological approaches. Improving access to such data creates easier on ramps to quantitative neuroimaging research.

As XNAT is the key format for image repositories, having Rxnat, an intuitive R interface and querying system native to R, can substantially accelerate this process. Rxnat can be used to assist existing researchers working in R and neuroimaging and lower the entry to computational neuroimaging in R.

The Rxnat package substantially simplifies querying and downloading images from XNAT repositories for researchers familiar with R. Rxnat complements PyXNAT/XNATpy and lowers the computational barrier for a large number of analysts using R. Given the ever increasing number of large imaging data sets, the importance of packages, such as Rxnat, is likely to increase. Indeed, the most important achievement of Rxnat is that it allows the incorporation of the XNAT data into image processing pipelines. This circumvents the need to use Web GUIs to manually download the data. Moreover, because the downloading process is now scripted, the approach substantially improves the reproducibility of a study by having explicit lines of code for inclusion/exclusion criteria as well as direct calls to publicly available image repositories.

For large scale studies, having a local image repository on a cluster is key for being able to generate results in a fast and secure way. Through the use of automated tasks, such as Unix cron jobs or the task scheduler for Windows systems, Rxnat can synchronize image repository with the upstream data and prepare it for *ad-hoc* analyses.

As part of the Neuroconductor project, Rxnat is an important upstream component of the processing and analysis of neuroimaging data. Indeed, Rxnat provides the connection with XNAT repositories, while Neuroconductor provides various interfaces to popular imaging software packages such as FSL, AFNI (https://afni.nimh.nih.gov/), MRICloud (https://mricloud.org/), and ANTs (http://stnava.github.io/ANTs/). As working with multiple R software packages software that are constantly changing can be difficult, we simplify the process by providing a Docker image for Neuroconductor (https://neuroconductor.org/docker-release). Thus, we aim to provide data access tools as well as analysis pipelines, ready for users. Coupled with rapidly developing tools for reproducible research in R, Neuroconductor and its packages, including Rxnat, are becoming a viable option for end-to-end analyses of neuroimaging data using modern best practices.

## Data Availability Statement

Publicly available datasets were analyzed in this study. This data can be found here: https://www.nitrc.org and https://db.humanconnectome.org.

## Ethics Statement

Ethical review and approval was not required for the study on human participants in accordance with the local legislation and institutional requirements. The patients/participants provided their written informed consent to participate in this study.

## Author Contributions

AG and JM wrote 90% of the manuscript and put together the example pipeline. AG was responsible for submitting the analysis to the cluster and aggregating the results. BC and CC oversaw the project, wrote the additional 10%, polished the manuscript, and managed the project. All authors contributed to the article and approved the submitted version.

## Conflict of Interest

The authors declare that the research was conducted in the absence of any commercial or financial relationships that could be construed as a potential conflict of interest.

## References

[B1] Achterberg H. (2015). XNATpy. Python Module version 0.3.24.

[B2] Burger M. Juenemann K. Koenig T. (2018). RUnit: R Unit Test Framework. R package version 0.4.32.

[B3] Fisher A. (2020). ggBrain: ggplot Brain Images. R package version 0.1.2.

[B4] Fortin J.-P. Parker D. Tunç B. Watanabe T. Elliott M. A. Ruparel K.. (2017). Harmonization of multi-site diffusion tensor imaging data. Neuroimage 161, 149–170. 10.1016/j.neuroimage.2017.08.047 28826946 PMC5736019

[B5] Gentleman R. C. Carey V. J. Bates D. M. Bolstad B. Dettling M. Dudoit S.. (2004). Bioconductor: open software development for computational biology and bioinformatics. Genome Biol. 5:R80. 10.1186/gb-2004-5-10-r80 15461798 PMC545600

[B6] Gorgolewski K. Burns C. D. Madison C. Clark D. Halchenko Y. O. Waskom M. L.. (2011). Nipype: a flexible, lightweight and extensible neuroimaging data processing framework in Python. Front. Neuroinform. 5:13. 10.3389/fninf.2011.00013 21897815 PMC3159964

[B7] Herrick R. Horton W. Olsen T. McKay M. Archie K. A. Marcus D. S. (2016). XNAT central: open sourcing imaging research data. Neuroimage 124(Pt B), 1093–1096. 10.1016/j.neuroimage.2015.06.076 26143202 PMC4965359

[B8] Jenkinson M. Beckmann C. F. Behrens T. E. J. Woolrich M. W. Smith S. M. (2012). FSL. Neuroimage 62, 782–790. 10.1016/j.neuroimage.2011.09.015 21979382

[B9] Kennedy D. N. Haselgrove C. Riehl J. Preuss N. Buccigrossi R. (2016). The NITRC image repository. Neuroimage 124, 1069–1073. 10.1016/j.neuroimage.2015.05.074 26044860 PMC4651733

[B10] Landman B. A. Ribbens A. Lucas B. Davatzikos C. Avants B. Ledig C. (2012). MICCAI 2012 Workshop on Multi-Atlas Labeling. CreateSpace Independent Publishing Platform.

[B11] Marcus D. S. Olsen T. R. Ramaratnam M. Buckner R. L. (2007). The Extensible Neuroimaging Archive Toolkit: an informatics platform for managing, exploring, and sharing neuroimaging data. Neuroinformatics 5, 11–34. 10.1385/NI:5:1:11 17426351

[B12] Mowinckel A. M. Piñeiro D. (2019). Visualisation of brain statistics with R-packages ggseg and ggseg3d. arXiv [Preprint]. arXiv:1912.08200.

[B13] Muschelli J. (2019). extrantsr: Extra Functions to Build on the ‘ANTsR’ Package. R package version 3.9.8.1.

[B14] Muschelli J. (2020). neurobase: ‘*Neuroconductor’ Base Package with Helper Functions for ‘nifti’ Objects*. R package version 1.29.0.

[B15] Muschelli J. Gherman A. (2020). papayaWidget: Embed an ‘Papaya’ Image Viewer. R package version 0.7.1.

[B16] Muschelli J. Gherman A. Fortin J. P. Avants B. Whitcher B. Clayden J. D. (2018). Neuroconductor: an R platform for medical imaging analysis. Biostatistics 20, 218–239. 10.1093/biostatistics/kxx068 PMC6409417 29325029

[B17] Muschelli J. Sweeney E. Lindquist M. Crainiceanu C. (2015). fslr: Connecting the fsl software with R. R J. 7, 163–175. 10.32614/RJ-2015-013 27330830 PMC4911193

[B18] R Core Team (2017). R: A Language and Environment for Statistical Computing. Vienna: R Foundation for Statistical Computing.

[B19] Reinhold J. C. Dewey B. E. Carass A. Prince J. L. (2019). “Evaluating the impact of intensity normalization on MR image synthesis,” in Medical Imaging 2019: Image Processing (International Society for Optics and Photonics). 10.1117/12.2513089 PMC6758567 31551645

[B20] Schwartz Y. Barbot A. Thyreau B. Frouin V. Varoquaux G. Siram A.. (2012). PyXNAT: XNAT in Python. Front. Neuroinform. 6:12. 10.3389/fninf.2012.00012 22654752 PMC3354345

[B21] Shinohara R. T. Sweeney E. M. Goldsmith J. Shiee N. Mateen F. J. Calabresi P. A.. (2014). Statistical normalization techniques for magnetic resonance imaging. Neuroimage Clin. 6, 9–19. 10.1016/j.nicl.2014.08.008 25379412 PMC4215426

[B22] Sievert C. (2020). Interactive Web-Based Data Visualization with R, Plotly, and Shiny. Chapman and Hall/CRC. 10.1201/9780429447273

[B23] Tabelow K. Whitcher B. (2011). Special volume on magnetic resonance imaging in r. J. Stat. Softw. 44, 1–6. 10.18637/jss.v044.i01

[B24] Temple Lang D. (2020). RCurl: General Network (HTTP/FTP/...) Client Interface for R. R package version 1.98-1.1.

[B25] Tustison N. J. Avants B. B. Cook P. A. Zheng Y. Egan A. Yushkevich P. A.. (2010). N4ITK: improved N3 bias correction. IEEE Trans. Med. Imaging 29, 1310–1320. 10.1109/TMI.2010.2046908 20378467 PMC3071855

[B26] Tustison N. J. Shrinidhi K. Wintermark M. Durst C. R. Kandel B. M. Gee J. C.. (2015). Optimal symmetric multimodal templates and concatenated random forests for supervised brain tumor segmentation (simplified) with ANTsR. Neuroinformatics 13, 209–225. 10.1007/s12021-014-9245-2 25433513

[B27] Van Essen D. C. Smith S. M. Barch D. M. Behrens T. E. Yacoub E. Ugurbil K. (2013). The WU-Minn human connectome project: an overview. Neuroimage 80, 62–79. 10.1016/j.neuroimage.2013.05.041 23684880 PMC3724347

[B28] Wang H. Suh J. W. Das S. R. Pluta J. B. Craige C. Yushkevich P. A. (2012). Multi-atlas segmentation with joint label fusion. IEEE Trans. Pattern Anal. Mach. Intell. 35, 611–623. 10.1109/TPAMI.2012.143 22732662 PMC3864549

[B29] Wickham H. (2011). testthat: get started with testing. R J. 3, 5–10. 10.32614/RJ-2011-002

[B30] Wickham H. (2016). ggplot2: Elegant Graphics for Data Analysis. New York, NY: Springer-Verlag 10.1007/978-3-319-24277-4_9

[B31] Wickham H. (2019). httr: Tools for Working with URLs and HTTP. R package version 1.4.1.

[B32] Wickham H. Francois R. Henry L. Muller K. (2019). dplyr: A Grammar of Data Manipulation. R package version 0.8.3.

[B33] Xie Y. (2014). “knitr: a comprehensive tool for reproducible research in R,” in Implementing Reproducible Computational Research, eds V. Stodden, F. Leisch, and R. D. Peng (Chapman and Hall/CRC).

[B34] Yarkoni T. Poldrack R. A. Nichols T. E. Van Essen D. C. Wager T. D. (2011). Large-scale automated synthesis of human functional neuroimaging data. Nat. Methods 8:665. 10.1038/nmeth.1635 21706013 PMC3146590

[B35] Zhang Y. Brady M. Smith S. (2001). Segmentation of brain MR images through a hidden Markov random field model and the expectation-maximization algorithm. IEEE Trans. Med. Imaging 20, 45–57. 10.1109/42.906424 11293691

[B36] Zuo X.-N. Anderson J. S. Bellec P. Birn R. M. Biswal B. B. Blautzik J.. (2014). An open science resource for establishing reliability and reproducibility in functional connectomics. Sci. Data 1:140049. 25977800 10.1038/sdata.2014.49 PMC4421932

