# Supplementary material for: Rxnat: An Open-Source R Package for XNAT-Based Repositories
Source: Front Neuroinform. 2020 Nov 9;14:572068. doi: 10.3389/fninf.2020.572068 (PMC7680896; doi:10.3389/fninf.2020.572068)
Supplement: Supplementary file 1 [file Data_Sheet_1.PDF]

Supplementary Material

1 SUPPLEMENTARY TABLES AND FIGURES

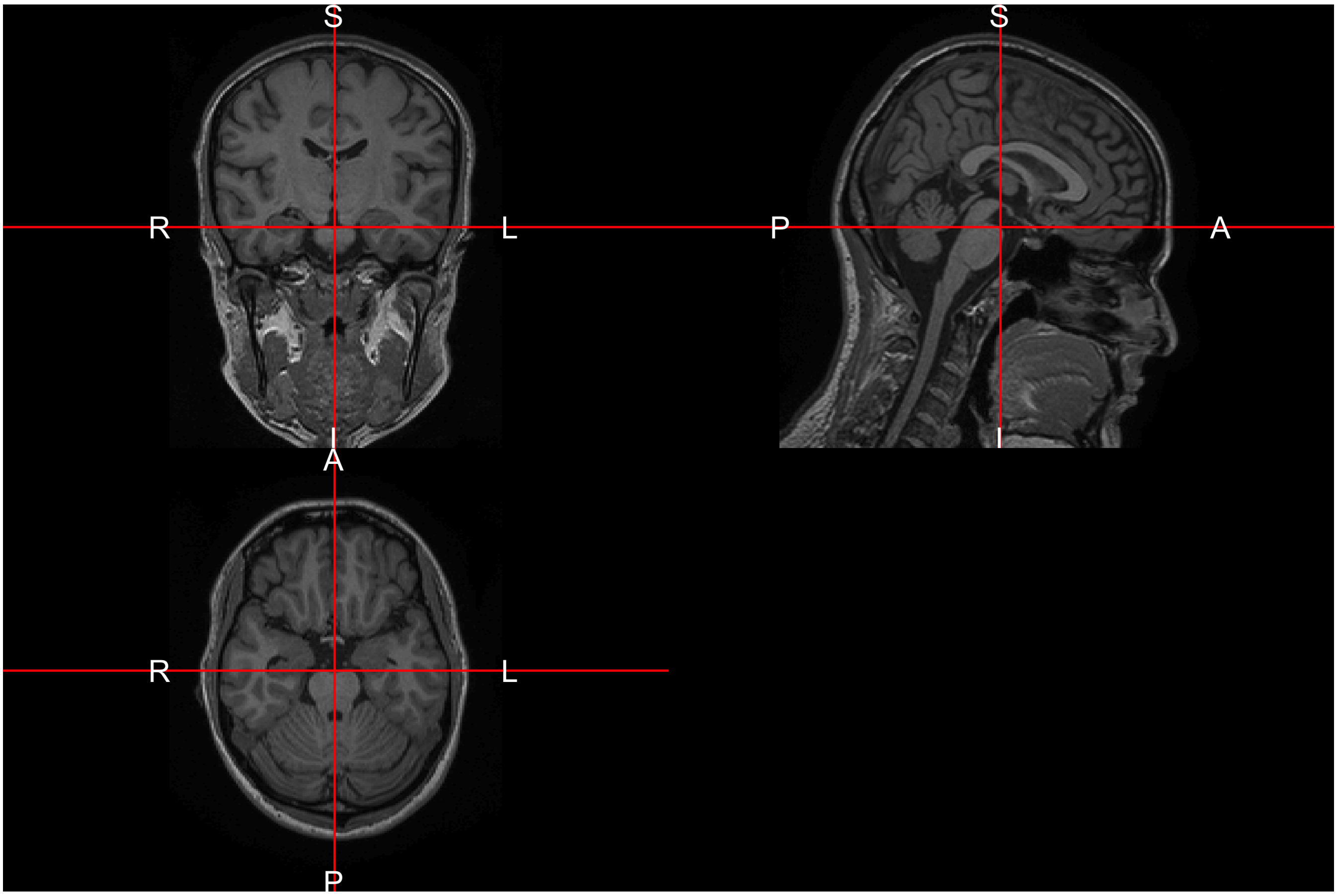

Figure S1. Original T1-weighted image for one study participant from the IXI database in NITRC. The letters indicate direction: P-posterior, A-anterior, S-superior, I-inferior, L-left, R-right.
